# Phytochemical analysis, antioxidant, α-glucosidase inhibitory activity, and toxicity evaluation of *Orthosiphon stamineus* leaf extract

**DOI:** 10.1038/s41598-023-43251-2

**Published:** 2023-10-09

**Authors:** Mustofa Ahda, Irwandi Jaswir, Alfi Khatib, Qamar Uddin Ahmed, Nurkhasanah Mahfudh, Yunita Dewi Ardini, Sharifah Nurul Akilah Syed Mohamad, Muslih Anwar, Hernawan Hernawan, Kazuo Miyashita, Ahmad Mohammad Salamatullah

**Affiliations:** 1 Department of Pharmaceutical Chemistry, Faculty of Pharmacy, Universitas Ahmad Dahlan, Yogyakarta, Indonesia; 2 Department of Pharmaceutical Technology, Faculty of Pharmacy, Universitas Ahmad Dahlan, Yogyakarta, Indonesia; 3 Department of Pharmaceutical Chemistry, Faculty of Pharmacy, International Islamic University Malaysia, Kuantan, Malaysia; 4 International Institute for Halal Research and Training (INHART), International Islamic University Malaysia, Kuala Lumpur, Malaysia; 5 Paediatric Dentistry and Dental Public Health Department, Kulliyyah of Dentistry, International Islamic University Malaysia, 25200 Kuantan, Pahang Malaysia; 6 Research Center for Food Technology and Process, National Research and Innovation Agency (BRIN), Yogyakarta, 55861 Indonesia; 7 Faculty of Fisheries Sciences, Hokkaido University, Sapporo, Japan; 8 Department of Food Science & Nutrition, College of Food and Agricultural Sciences, King Saud University, 11451 Riyadh, Saudi Arabia

**Keywords:** Biochemistry, Drug discovery

## Abstract

*Ocimum aristatum*, commonly known as *O. stamineus*, has been widely studied for its potential as an herbal medicine candidate. This research aims to compare the efficacy of water and 100% ethanolic extracts of *O. stamineus* as α-glucosidase inhibitors and antioxidants, as well as toxicity against zebrafish embryos. Based on the study findings, water extract of *O. stamineus* leaves exhibited superior inhibition activity against α-glucosidase, ABTS, and DPPH, with IC_50_ values of approximately 43.623 ± 0.039 µg/mL, 27.556 ± 0.125 µg/mL, and 95.047 ± 1.587 µg/mL, respectively. The major active compounds identified in the extract include fatty acid groups and their derivates such as linoleic acid, α-eleostearic acid, stearic acid, oleanolic acid, and corchorifatty acid F. Phenolic groups such as caffeic acid, rosmarinic acid, 3,4-Dihydroxybenzaldehyde, norfenefrine, caftaric acid, and 2-hydroxyphenylalanine and flavonoids and their derivates including 5,7-Dihydroxychromone, 5,7-Dihydroxy-2,6-dimethyl-4H-chromen-4-one, eupatorin, and others were also identified in the extract. Carboxylic acid groups and triterpenoids such as azelaic acid and asiatic acid were also present. This study found that the water extract of *O. stamineus* is non-toxic to zebrafish embryos and does not affect the development of zebrafish larvae at concentrations lower than 500 µg/mL. These findings highlight the potential of the water extract of *O. stamineus* as a valuable herbal medicine candidate, particularly for its potent α-glucosidase inhibition and antioxidant properties, and affirm its safety in zebrafish embryos at tested concentrations.

## Introduction

The utilization of herbal plants for treating various acute ailments, including diabetes, has seen a significant increase in recent times. Studies indicate that over 80% of people worldwide have resorted to herbal medicine to enhance their immune systems^1^. Herbal remedies have demonstrated positive effects in managing mild to moderate diseases^2^. Consequently, screening techniques are essential to identify potent herbal candidates for anti-diabetic drugs.

In Asia, the leaves of *Ocimum stamineus*, commonly known as *O. stamineus*, have been recognized for their potential in diabetes treatment. The leaves contain flavonoids and their derivatives, including sinensetin, which exhibit disease-prevention properties^3,4^. According to Ahda et al.^5^, this plant has various mechanisms of action to lower blood glucose levels, including boosting GLP-1 secretion and blocking α-glucosidase and α-amylase. Additionally, a nuvastatic supplement made from standardized *O. stamineus* extract for administration in diabetic retinopathy (DR) patients in clinical research has been registered (registration number NCT04552600).

The efficacy and potency of herbal medicines have made them a popular preventive measure against various diseases, with fewer risks of side effects compared to synthetic drugs. It is crucial to conduct toxicity evaluations to ensure the safety of herbal remedies. Improper usage, high dosage, long-term consumption, and inadequate monitoring can lead to increased side effects and potential toxicity^6–8^. Therefore, toxicological assessments are necessary to ensure the safety of potent herbal candidates.

Standard toxicity evaluations typically involve clinical studies on humans, as they provide relevant data for assessing the safety of herbal medicines before market authorization^8^. However, prior to human testing, it is essential to conduct preliminary screening tests to assess for toxic herbs. These tests commonly employ animal models such as mice and rabbits. This study aimed to identify the non-toxic potent extracts of *O. stamineus* leaves through toxicity evaluation using zebrafish embryos.

This method for toxicity evaluation offers several advantages, including large sample size, short-term use, genetic similarity, and cost-effectiveness^9,10^. Previous studies have assessed the toxicity of various herbal extracts on zebrafish embryos, revealing lethal concentrations (LC_50_) dependent on the type of herbal and solvent used. For instance, the LC_50_ values for water–methanol and water–ethanol extracts of *Moringa oleifera* were found to be 163.87 ± 12.88 mg/mL and 337.48 ± 30.04 mg/mL, respectively^11^. Norazhar et al.^12^ demonstrated an LC_50_ value of 419.84 μg/mL for the methanolic extract of *Christia vespertilionis*.

These findings highlight the significant impact of the type of herb and extraction solvent used on final toxicity. Notably, Sajak et al.^13^ compared toxicity testing using zebrafish embryos and Wistar rats, finding that a polyphenolic-rich herbal mixture (PRM) had an LC_50_ of approximately 487.50 μg/mL in zebrafish embryos, despite no lethal effects being observed in rats at a dose of 2000 mg/kg body weight. Zebrafish embryos exhibited higher sensitivity in toxicity evaluations, allowing for more sensitive detection and for herbal extracts with LC_50_ values above 500 μg/mL to be classified as non-toxic. Therefore, this research provides an original viewpoint before approval and use of this herb in future clinical studies and treatment.

## Material and methods

### Samples preparation

*O. stamineus* was planted by the civil society in Yogyakarta, Indonesia (East longitude: 107° 15′ 03″ and East longitude: 107° 29′ 30″; South latitude: 7° 34′ 51″ and 7° 47′ 30″). Time of cultivation: February–March 2021 (Condition: last rainy season to first dry season). Because this plant grows naturally, it can be used freely (no permission required). Taxonomic identification was performed by Hery Setiyawan, M.Si (Department of Biology, Universitas Ahmad Dahlan). Fresh samples (leaf and stem) were washed and dried in an oven at 45 °C for four days. The dried samples were then ground into a powder and separated with a sieve size of 60 mesh. All procedures followed Good Agricultural and Collection Practice (GACP) scientific guidelines for starting materials of herbal origin and legislation.

### Extraction process

The leaf powder of *O. stamineus* was extracted using ultrasonic-assisted extraction. 10 g of *O. stamineus* leaf powder was dissolved in solvent (100% ethanol and water) with a solid-to-solvent ratio of 1:10 (w/v). Both samples were sonicated for 60 min at 50 °C using an ultrasonic batch and were incubated overnight^14^. Filtration was used to obtain the extract solution, which was then evaporated to obtain the dried extract. All extracts were freeze-dried and stored at 4 °C for further testing.

### Determination of total phenolic content

Total phenolic content (TPC) was determined using the Folin Ciocalteu method, as described by Ahda et al.^15^. 25 mg extract of *O. stamineus* leaf or stem was dissolved in 25 mL Aquadest and mixed with 1.5 mL Folin Ciocalteu (1:10 in water) for 3 min. The solution was then mixed with 1.2 mL of 7.5% sodium carbonate (w/v) and then left for 60 min. Absorbance was measured at 743 nm using a UV–Vis spectrophotometer (Shimadzu Uv–Vis 1800, Japan). Gallic acid equivalent concentration was used as the standard for total phenolic concentration. Phenolic content was expressed in μg/mg GAE (gallic acid equivalent) of dry weight extract. All measurements were carried out in triplicates.

### Determination of total flavonoid content

Total flavonoid content (TFC) was determined by colorimetric method using aluminum chloride (AlCl_3_) as reported by Chandra et al.^16^ with minor modifications. 25 mg of ethanolic extracts of *O. stamineus* leaf or stem was dissolved in 25 mL ethanol. 1 mL of the solution was mixed with 0.5 mL of 10% AlCl_3_ and incubated at room temperature for 74 min. The absorbance was measured at 410 nm with a Uv–Vis spectrophotometer (Shimadzu Uv–Vis 1800, Japan). Quercetin standard was measured ranging from 5 to 20 µg/mL as the standard for total flavonoid concentration. TFC was calculated as μg/g quercetin equivalent (QE) of dried extract. All measurements were carried out in triplicates.

### Inhibition activity of DPPH (2,2-diphenyl-1-picrylhydrazyl) radicals

25 mg of *O. stamineus* leaf or stem was dissolved in 25 mL ethanol. The extract samples were diluted to concentrations ranging from 0 to 500 µg/mL^15^. 1 mL of extract solution was mixed with 1 mL of 0.05 mM DPPH solution and vortexed for 1 min. The mixture was kept for one hour. The mixture absorbance was analyzed using a UV–Vis spectrophotometer at 516 nm (Shimadzu Uv–Vis 1800, Japan). The 50% inhibition concentration (IC_50_) was calculated following the equation below:

$$\mathrm{\%\,\, Inhibition \,\,Activity}=\left(\frac{{\mathrm{A}}_{0}-{\mathrm{A}}_{1}}{{\mathrm{A}}_{0}}\right) \times 100\%,$$

where A_0_ is the absorbance of the control, A_1_ is the absorbance of the samples.

### Inhibition activity of ABTS (2,2-Azino-bis(3-ethylbenzothiazoline-6-sulfonic acid) radicals

The inhibition activity of ABTS radicals was analyzed using a slightly modified method previously described by Byun et al.^17^. The reagent was incubated for 24 h at 37 °C after being mixed with 7.4 mM ABTS and 2.45 mM potassium persulfate solution in a 1:1 (v/v) ratio. The ABTS working solution was ready for use when absorbance value = 0.70 ± 0.02 at 734 nm. Briefly, 1 mL of ABTS solution was incubated with 1 mL of extract for 74 min. The solution used as a controlled standard is quercetin. ABTS radical scavenging activity was calculated using the following equation:

$$\mathrm{\%\,\, Inhibition \,\,Activity}=\left(\frac{\mathrm{A}-\mathrm{B}}{\mathrm{A}}\right) \times 100\%,$$

where A is the absorbance of the control, B is the absorbance of the test sample.

### Determination of inhibition activity against α-glucosidase enzyme

Inhibition activity of *O. stamineus* extract was determined following slightly modified methods described by^18^. α-glucosidase enzyme was prepared in a sodium phosphate buffer with a pH of 6.8 (15 U/100 mL). *O. stamineus* extract (final concentration ranging betwen 10–100 µg/mL) was reacted with α-glucosidase for 15 min. After that, the solution was incubated for 20 min with 5 mM p-nitrophenyl α-glucopyranoside (pNPG). The final composition ratio of α-glucosidase: pNPG extract was 200 µL:200 µL:200 µL. Finally, 1 mL of 0.2 M Na_2_CO_3_ was added to break up the reaction. All solutions were analysed using a UV–Vis spectrophotometer (Shimadzu Uv–Vis 1800, Japan) at 400 nm. Percentage inhibition was determined by the equation:

$$\mathrm{\%\,\, Inhibition\,\, Activity}=100 \times (1-\left(\frac{{\mathrm{A}}_{\mathrm{s}}-{\mathrm{A}}_{\mathrm{b}}}{{\mathrm{A}}_{\mathrm{c}}}\right),$$

where As : Absorbance of sample, Ab: Absorbance of blank (without enzyme), Ac: Absorbance of control (DMSO + enzyme + PNPG).

### Chemical analysis of *O*. *stamineus* leaf extract using high-resolution mass spectrometry (HRMS)

Samples were prepared following Windarsih et al.^19^ with slight modifications. Analysis was performed by adding LC–MS-grade methanol into *O. stamineus* leaf extracts (water and ethanol extract). The mixture was vortexed for 2 min before being subjected to 30 min of ultrasonication. The pellet and the supernatant were separated by centrifuging at 5000 rpm for 5 min. The supernatant was transferred to a 2 mL HPLC vial after being filtered with a 0.22 m PTFE filter.

Analysis was performed using a Thermo Scientific Vanquish UHPLC system with a binary pump coupled with high-resolution mass spectrometry Q-Exactive Orbitrap. Separation was performed on a Thermo Scientific™ Acclaim™ VANQUISH™ C18 stationary phase column with the particle size in column dimensions (150 mm 2.1 mm ID 2.2 m), LC–MS-grade water (Merck) containing 0.1% formic acid (A) and LC–MS-grade methanol (Merck) containing 0.1% formic acid (B) as mobile phase. 10 µL of the sample was injected into the column with a flow gradient of 0.30 mL/min from 5 to 90% B in 20 min and maintained at 95% A for 5 min. For mass spectrometric conditions, the sheath gas flow rate was set at 32 arbitrary units (AU), while the auxiliary and sweep gas flow rates were set at 8 and 4 AU, respectively. Scanning was carried out in both MS1 and MS2, with MS1 having a resolution of 70,000 and MS2 having a resolution of 17,500. Analysis was carried out concurrently in positive and negative ionization modes, with the collision energy set at 10 eV and the analytes scanned in the range of 66.7–1000 m/z. The chemical compositions of untargeted and targeted metabolites were identified using Compound Discoverer 3.2 software. The compounds were then examined for peak extraction using MzCloud and ChemSpider databases, with annotated masses ranging from -5 ppm to 5 ppm. Only chemicals with a complete MzCloud and ChemSpider match were selected for analysis. Peak intensities were modified to represent the overall spectrum intensity.

### Toxicity evaluation using zebrafish embryos

Toxicity testing was performed following the OECD test guideline (TG) 236 described by Nipun et al.^20^ with slight modifications. This procedure has been approved by the IIUM ethics committee, namely the IIUM Animal Care and Use Committee (I-ACUC) with register number: IACUC 2022-018. Zebrafish AB strain eggs (age < 5 h) were used in this study. 100% ethanolic and water extracts of *O. stamineus*, negative control (E3 medium), positive control (4 mg/L of 3,4-dichloroaniline in E3 medium), and solvent control (1% dimethyl sulfoxide in E3 medium) were used in this investigation. Each group contained 20 eggs for each test concentration and 4 eggs as the internal plate control for each plate. Each well contained 300 μL of the solution consisting of 150 μL of E3 medium and 150 μL of the sample in 1% dimethylsulfoxide (DMSO). Zebrafish deaths were counted at intervals of 24 h, 48 h, 72 h, and 96 h. LC_50_ was calculated via zebrafish mortality. Besides toxicity, teratogenicity criteria such as the frequency and severity of morphological abnormalities and hatching rate were also recorded. All methods are reported in accordance with Arrive guidelines.

### Data analysis

The data was expressed as mean ± standard deviation (SD). One-way analysis of variance (ANOVA) was performed; significant values were set at confidence intervals of up to 95% and p < 0.05.

### Ethics approval and consent to participate

Ethics approval granted by IIUM under approval no. IACUC 2022-018.

## Results and discussion

### Yields, total phenolic content (TPC), and total flavonoids content (TFC) of O. stamineus extracts

The extraction process of herbs is an important factor in the industry, due to the need for producing high yields while retaining bioactivity. Extracted herbs typically yield around 33.69 wt%, 6.05%, 4.42%, and 3.08% using various solvents such as water, ethyl acetate, ethanol, and n-hexane, respectively^21^. The results of this study show that different solvents used in extraction affect final yields. This is consistent with the findings of Ghasemzadeh et al.^22^, who report that increasing solvent polarity tends to increase yields. Extraction using 100% ethanol had no significant effect on yields between the leaf and stem of *O. stamineus*, whereas water extraction of *O. stamineus* leaves produced higher yields compared to stems.

Furthermore, *O. stamineus* water extract contained more TPC than other extracts. The highest concentration of TFC was found in 100% ethanolic extract of *O. stamineus* leaves (See Table 1). According to Ibrahim and Jaafar^23^, *O. stamineus* leaves contained around 3.11 ± 0.27 mg/g and 1.47 ± 0.21 mg/g of TPC and TFC, respectively. Meanwhile, *O. stamineus* stems contained less TPC and TFC than its leaves. Therefore, the antioxidant activity of *O. stamineus* leaves is predicted to be greater than its stem. Hence, the assessments of α-glucosidase inhibitory activity, antioxidant properties, and toxicity on zebrafish embryos were performed using 100% ethanolic and water extracts of *O. stamineus* leaves.

**Table 1:** Yields, TPC, and TFC of *O. stamineus* extracts.

*O. stamineus* extract	Yield (%)	Total phenolic content (µg/mg)	Total flavonoid content (µg/mg)
Leaf			
100% ethanol	7.009 ± 0.536^a^	18.28 ± 0.649^a^	49.07 ± 0.144^a^
Water	13.45 ± 0.890^b^	84.37 ± 0.351^b^	5.066 ± 0.032^b^
Stem			
100% ethanol	6.214 ± 0.754^a^	16.10 ± 0.595^c^	10.81 ± 0.582^c^
Water	2.709 ± 0.385^c^	75.33 ± 1.078^d^	4.301 ± 0.048^d^

n = 3, Tukey’s test, p < 0.05.

^a-d^ mean Values with different alphabet are significantly different at P < 0.05.

### *O*.* stamineus* leaf extract as α-glucosidase inhibitors and antioxidants

*O. stamineus* leaves were previously reported for their anti-diabetes, antioxidant, and anti-inflammation activities, as reported by Wang et al.^24^. Therefore, the goal of this study is to assess the activity of 100% ethanolic extract and water extract of *O. stamineus* leaves as antioxidants and α-glucosidase inhibitors. In a previous study, isolated sinensetin from this herb inhibited α-glucosidase (IC_50_ ~ 0.66 mg/ml), while crude extract had an IC_50_ of 4.63 mg/ml^25^. To reduce the activity of α-glucosidase, sinensetin binds to the polar residues (Arg194, Ser343, Asp450, Glu443, Cys447, Tyr340, Gln220, Glu339, Ser453) and hydrophobic residues (Ala341, Pro338, Pro446, Val342, Trp213) of the molecule^26^.

Table 2 shows that 100% ethanolic extract had α-glucosidase inhibition activity compared to the water extract. According to a previous study, IC50 of the methanolic and ethanolic extracts of *O. stamineus* leaves were in the upper range of 1250 ppm^27^. Due to its ability to inhibit ABTS and DPPH radicals, the *O. stamineus* leaf water extract can be used as an antioxidant. The antioxidant capacity of this herb measured using Oxygen Radical Absorbance Capacity (ORAC) and DPPH methods were 65.21 ± 2.41 µmol Trolox equivalent/g and µmol Trolox equivalent/g, respectively^23^.

**Table 2:** Inhibition activity of *O. stamineus* leaf extracts towards α-glucosidase, DPPH, and ABTS.

*O. stamineus* extract	α-glucosidase inhibition IC_50_ (µg/mL)	ABTS inhibition IC_50_ (µg/mL)	DPPH inhibition IC_50_ (µg/mL)
100% ethanol	> 150	64.03 ± 0.108^a^	134.0 ± 0.099^a^
Water	43.62 ± 0.039^a^	27.56 ± 0.125^b^	95.05 ± 1.587^b^
Quercetin	20.23 ± 0.890^b^	2.209 ± 0.002^c^	2.215 ± 0.029^c^

n = 3, Tukey’s test, p < 0.05.

^a-c^ mean Values with different alphabet are significantly different at P < 0.05.

The active components present in the *O. stamineus* leaf, including sinensetin, contributes to the extract’s potency^25^. According to Yam et al.^28^, increasing the polarity of the solvent used reduces sinensetin, 3′hydroxy-5,6,7,4′-tetramethoxyflavone, and Eupatorin content in the extract. However, the water extract of *O. stamineus* leaves has high potency as an α-glucosidase inhibitor and antioxidant agent, as reported in Table 2. The difference in biological activities between the two extracts may be influenced by other active compounds present. Therefore, this study uses HR-MS to identify chemical compounds that are active in these extracts.

### Toxicity evaluation of *O*. *stamineus* extracts using zebrafish embryos

Herbal remedies are currently preferred in the medical sector. However, their effectiveness, efficiency, and safety are crucial consideration factors before use in treatment. Zebrafish embryos are a frequently used model to test for the toxicity of herbs. Their use in pre-clinical studies provides the link between in-vitro and in-vivo studies^29^. Zebrafish embryo toxicity (ZFET) testing offers a number of advantages, including the large sample size, low cost, and simple handling^9,30^. Therefore, this toxicity evaluation model was used in this study.

**Figure 1:**
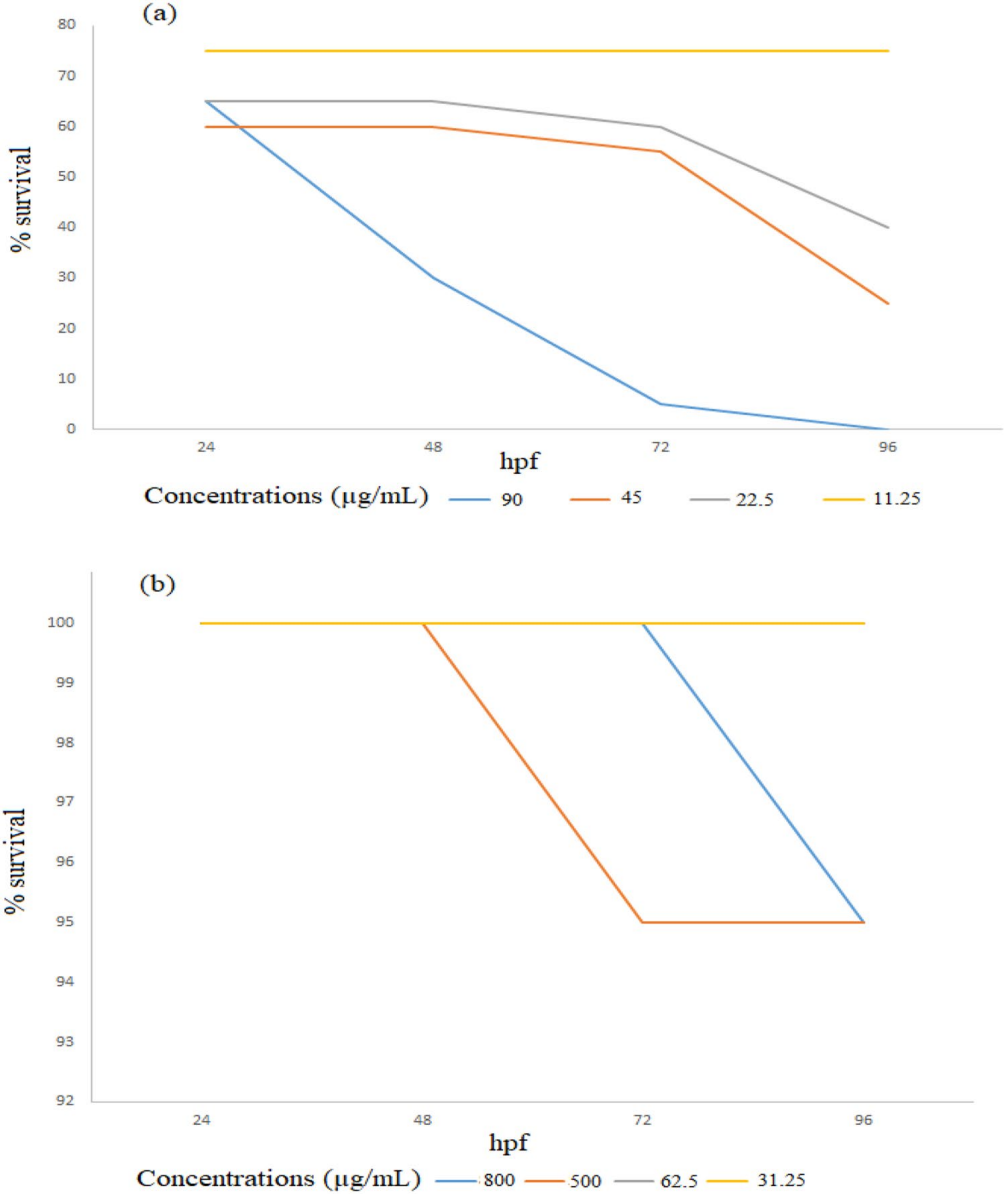
Percentage survival of zebrafish larvae during 96 hpf. (**a**) 100% ethanolic extract of *O. stamineus* leaves and (**b**) water extract of *O. stamineus* leaves.

**Figure 2:**
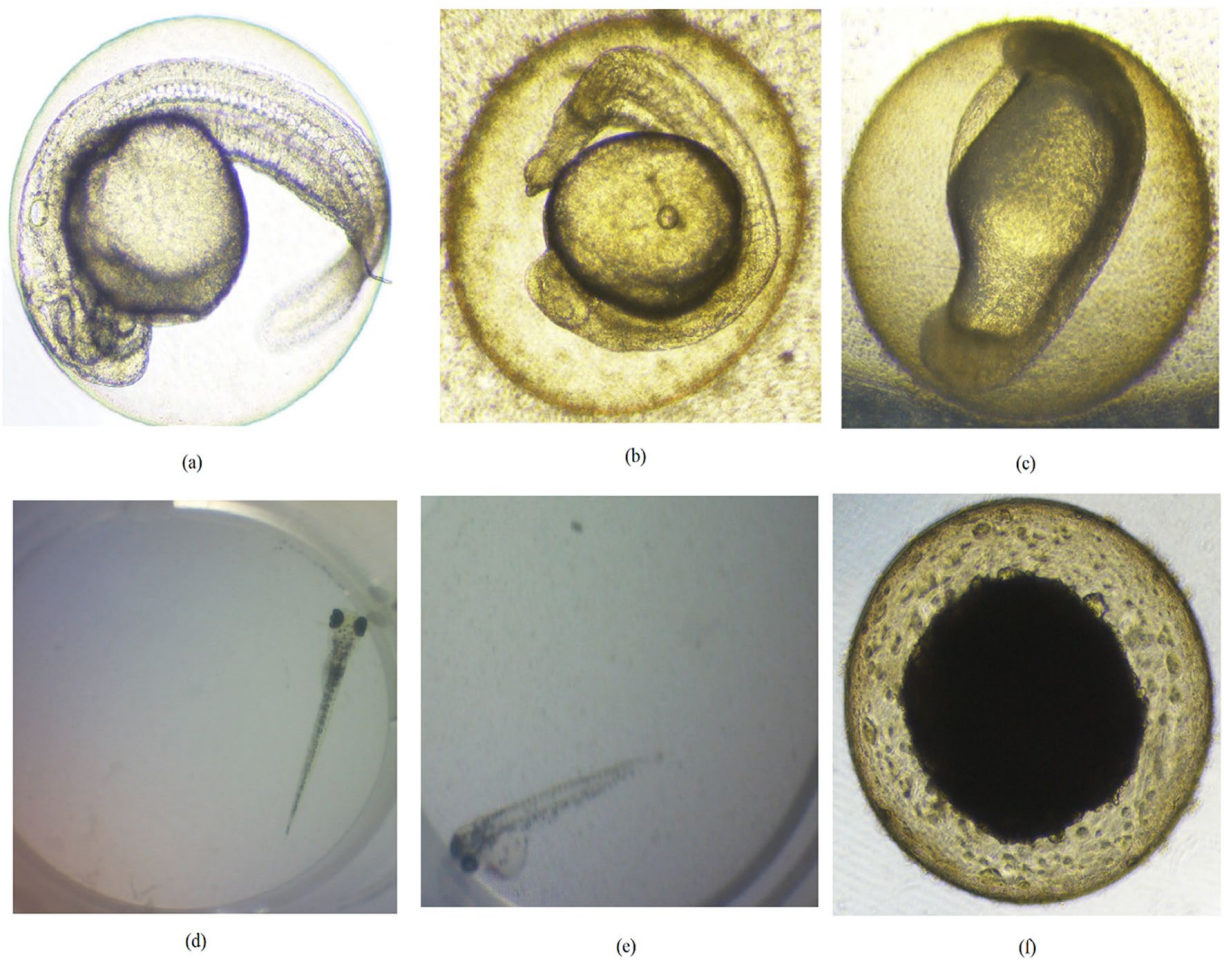
The development of zebrafish larvae (**a**–**c**) 24 hpf; (**d**–**f**) 96 hpf. (**a**,**e**) solvent control; (**b**,**e**) water extract of *O. stamineus* leaves at 500 µg/mL; (**c**,**f**) 100% ethanolic extract of *O. stamineus* extract at 90 µg/mL.

According to Table 3, 100% ethanolic extract of *O. stamineus* leaves had a worse effect on Zebrafish embryo development than water extract when concentration was below 100 g/mL. The survival rate of zebrafish larvae exposed to 100% ethanolic *O. stamineus* leaf extract at 22.5 µg/mL was less than 50%, while the survival rate of zebrafish larvae contacted by water extract at 800 µg/mL was still higher than 90% (Fig. 1). Less pigmentation, delayed hatching, yolk edema, heart edema, and crooked backbone were among the physical defects present in numerous zebrafish embryos (see Fig. 2).

**Table 3:** The effect of *O. stamineus* leaf extract on zebrafish embryo development for 96 hpf.

*O. stamineus* extract	Concentration applied (µg/mL)	Hatching defect	Less pigmentation	Yolk edema	Heart edema	Crooked backbone
100% ethanolic	90.0	+	+	+	+	+
	45.0	+	+	+	+	+
	22.5	–	–	–	–	–
	11.3	–	–	–	–	–
Water extract	800	+	+	+	+	+
	500	+	+	+	+	–
	250	–	–	–	–	–
	125	–	–	–	–	–
	62.5	–	–	–	–	–
Control	PC	+	+	+	+	+
	NC	–	–	–	–	–
	SC	–	–	–	–	–

*PC* positive control (4 mg/L of 3,4- dichloroaniline in E3 medium), *NC* negative control (E3 medium), *SC* solvent control (1% dimethyl sulfoxide in E3 medium).

Lethal concentration 50 (LC_50_) of the 100% ethanolic extract and aqueous extracts of *O. stamineus* leaves was 21.623 µg/mL and > 800 µg/mL, respectively. According to prior work by Ismail et al.^31^, the water extract of *O. stamineus* has an LC_50_ of 1685 µg/mL, therefore the results reported here are consistent with previous findings. According to the recommendations of the Organization for Economic Cooperation and Development, compounds with LC_50_ values of between 400 and 1000 µg/mL are classified as non-toxic^12,32^. In light of this research, *O. stamineus* leaf water extract is classified as a non-toxic herbal preparation.

### Chemical compound profiling of *O*. *stamineus* leaf extract

The identification of active compounds in herbs is an important aspect of quality control. Storage condition is an important factor in maintaining consistent herb quality. This study employs high-resolution mass spectrometry (HR-MS) to identify the active compounds in *O. stamineus* leaf extracts. This analytical method predicts the active compounds in complex samples, especially herbal plants. He et al.^33^, utilised HR-MS to identify 167 illegal medicines found in herbal tea. Additionally, 68 compounds from *B. intermedia* and 81 compounds from *S. marginata* have been detected using HR-MS^34^. HR-MS combined with chemometrics is more efficient for investigating herbs used in Traditional Chinese Medicine based on quality markers^35^.

**Figure 3:**
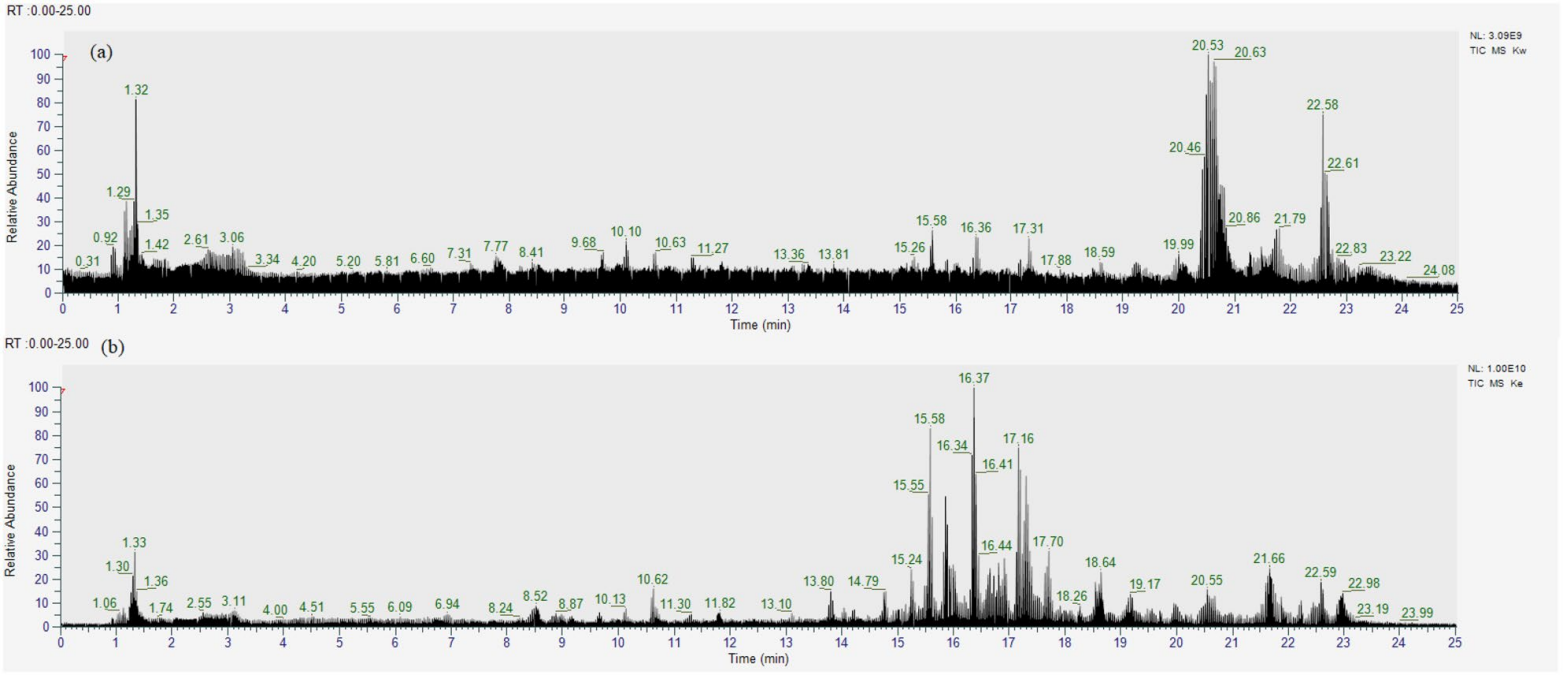
Mass spectrum of *O. stamineus* leaf extract: (**a**) water extract; (**b**) 100% ethanolic extract.

The HR-MS chromatograms show that water extract and 100% ethanolic extract of *O. stamineus* leaves contain different putative active compounds. According to Fig. 3, the water extract of *O. stamineus* leaves has dominant active compounds at retention times ranging between of 1–1.5 min and 20.5–23.5 min. These chemical compounds are grouped into fatty acid groups, triterpenoids, flavonoids and their derivates, quinones, hexoses compounds, phenolic compounds, and carboxylic acid groups and their derivates (Table 4). These compounds may explain the biological activities observed, including α-glucosidase inhibition and antioxidant activity. The extract also contains fatty acids such as stearic acid, α-eleostearic acid, linoleic acid, and others. Meanwhile, the 100% ethanolic extract contains carboxylic acid groups, flavonoid methyl ester groups, fatty acid groups, phenolic compound groups, and acyl groups. Table 4 lists the other active compounds present in this extract.

**Table 4:** The chemical structure formula of *O. stamineus* leaf extract.

Retention time (min)	Compounds	Structure	Annotation DeltaMass [ppm]	MS experiment	Area, 10^8^	
					WE	EE
1.179	d-Glucosamine	C_6_ H_13_NO_5_	− 1.76	179.07906	5.158	0.762
1.390	Gluconic acid	C_6_ H_12_O_7_	− 3.39	196.05764	1.207	2.567
1.895	Nicotinic acid	C_6_ H_5_NO_2_	− 0.48	123.03197	2.681	2.106
2.101	Citric acid	C_6_ H_8_ O_7_	− 3.64	192.02630	0.038	1.895
2.399	5-Deoxy-5-aminoshikimic acid	C_7_H_11_ N O_4_	− 3.05	173.06828	0.478	14.022
3.680	6-Oxo-pipecolinic acid	C_6_ H_9_NO_3_	− 2.73	143.05785	2.387	0.952
5.150	Homogentisic acid	C_8_ H_8_ O_4_	− 4.42	168.04152	2.179	0.595
5.194	L-Dopa	C_9_ H_11_NO_4_	− 1.30	197.06855	0.819	0.303
5.380	3-(2,4,5-Trihydroxyphenyl)propanoic acid	C_9_H_10_O_5_	− 3.16	198.05220	1.834	2.528
5.635	cis-zeatin	C_10_H_13_N_5_O	− 0.08	219.11199	1.503	0.761
5.861	Isovanillic acid	C_8_H_8_O_4_	− 0.67	168.04215	0.509	0.157
6.103	NP-012551	C_8_ H_8_O_4_	− 3.41	168.04168	0.779	0.499
6.132	4,5,7-Trihydroxycoumarin	C_9_ H_6_O_5_	− 0.90	194.02135	0.081	0.303
6.239	Kojic acid	C_6_H_6_O_4_	− 0.40	142.02655	1.444	0.070
6.841	DL-4-Hydroxyphenyllactic acid	C_9_ H_10_O_4_	− 4.33	182.05712	2.937	1.863
6.910	3,4-Dihydroxybenzaldehyde	C_7_H_6_O_3_	− 0.88	138.03157	3.441	0.072
7.142	2,3-Dihydroxy-2-(4-hydroxy-3-methoxybenzyl)succinic acid	C_12_H_14_O_8_	− 0.29	286.06879	0.175	0.192
7.256	Fraxetin	C_10_H_8_O_5_	2.64	208.03772	0.161	0.009
7.563	Salicylic acid	C_7_H_6_O_3_	− 0.88	138.03157	0.601	0.020
7.601	cis-caffeic acid	C_9_H_8_O_4_	− 4.20	180.04150	0.180	0.001
7.668	Methyl 4-hydroxycinnamate	C_10_H_10_O_3_	− 0.79	178.06285	0.001	0.225
7.868	Chlorogenic acid	C_16_H_18_O_9_	− 0.50	354.09490	0.357	0.096
8.354	5,7-Dihydroxychromone	C_9_H_6_O_4_	− 2.87	178.02610	3.335	0.602
8.378	3-Amino-5-hydroxybenzoic acid	C_7_H_7_NO_3_	− 0.72	153.04248	0.395	0.018
8.527	Kynurenic acid	C_10_H_7_NO_3_	− 1.90	189.04223	1.226	1.708
8.669	Trans-caffeic acid	C_9_H_8_O_4_	− 0.72	180.04213	32.337	2.173
8.670	(DL)-3-O-Methyldopa	C_10_H_13_NO_4_	− 2.31	211.08397	13.139	3.059
8.842	Vanillic acid	C_8_H_8_O_4_	− 4.96	168.04143	0.137	0.012
8.935	(6E)-6-(3,4-Dihydroxybenzylidene)-4-(3,4-dihydroxyphenyl)-3-hydroxy-2H-pyran-2,5(6H)-dione	C_18_H_12_O_8_	− 0.12	356.05318	0.017	0.083
9.227	3-Hydroxy-5-methyl-L-tyrosine	C_10_H_13_NO_4_	0.01	211.08446	0.536	0.101
9.310	Vanillin	C_8_H_8_O_3_	− 0.76	152.04723	0.957	0.011
9.415	Phaseolic acid	C_13_H_12_O_8_	− 0.53	296.05306	0.091	0.242
9.665	Pinostrobin	C_16_H_14_O_4_	− 0.09	270.08918	0.272	0.278
9.765	N-acetyldopamine	C_10_H_13_NO_3_	− 1.74	195.08920	0.689	0.059
10.091	coniferyl aldehyde	C_10_H_10_O_3_	− 4.24	178.06224	0.179	0.006
10.096	Caftaric acid	C_13_H_12_O_9_	− 0.06	312.04811	0.107	0.552
10.220	Norfenefrine	C_8_H_11_NO_2_	− 0.85	153.07885	3.736	0.693
10.222	2-Hydroxyphenylalanine	C_9_H_11_NO_3_	1.21	181.07411	3.412	0.674
10.318	Melevodopa	C_10_ H_13_NO_4_	− 3.05	211.08381	0.266	0.028
10.758	N-Aceyl-l-tyrosine	C_11_H_13_NO_4_	1.10	223.08470	0.031	3.423
10.843	Vanillylamin	C_8_H_11_NO_2_	− 1.08	153.07881	0.784	0.001
11.177	4-(3,4-dihydroxyphenyl)-7-hydroxy-5-{[(2S,3R,4S,5S,6R)-3,4,5-trihydroxy-6-(hydroxymethyl)oxan-2-yl]oxy}-2H-chromen-2-one	C_21_H_20_O_11_	− 0.80	448.10020	0.521	0.002
11.238	2-(3,4-Dihydroxyphenyl)-5,7-dihydroxy-4-oxo-4H-chromen-3-yl 6-O-β-d-xylopyranosyl-β-d-glucopyranoside	C_26_H_28_O_16_	− 0.76	596.13728	0.278	0.033
11.324	Umbelliferone	C_9_H_6_O_3_	− 0.37	162.03163	0.006	0.120
11.666	Quercetin-3β-d-glucoside	C_21_H_20_O_12_	0.26	464.09559	0.956	0.003
11.855	Ferulic acid	C_10_H_10_O_4_	− 3.40	194.05725	0.675	0.022
11.931	Rosmarinic acid	C_18_H_16_O_8_	− 0.54	360.08432	11.77	2.250
12.070	4-Anisic acid	C_8_H_8_O_3_	− 0.76	152.04723	0.210	0.028
12.451	5,7-Dihydroxy-2-(4-hydroxyphenyl)-4-oxo-4H-chromen-3-yl 6-O-(6-deoxyhexopyranosyl)hexopyranoside	C_27_H_30_O_15_	− 0.43	594.15822	0.151	0.004
12.546	Azelaic acid	C_9_H_16_O_4_	− 1.22	188.10463	5.180	1.798
12.862	5-Pentylresorcinol	C_11_H_16_O_2_	− 2.09	180.11465	1.402	0.101
13.104	Isorhamnetin	C_16_H_12_O_7_	− 0.39	316.05818	0.355	0.003
13.610	Quercetin	C_15_H_10_O_7_	− 0.63	302.04246	0.812	0.118
13.697	4-Methoxycinnamic acid	C_10_H_10_O_3_	− 0.97	178.06282	0.444	0.026
13.968	5,7-Dihydroxy-2,6-dimethyl-4H-chromen-4-one	C_11_H_10_O_4_	− 2.94	206.05730	5.977	0.351
14.019	Luteolin	C_15_H_10_O_6_	− 0.72	286.04753	1.427	0.073
14.171	Aurantioobtusin	C_17_H_14_O_7_	− 0.53	330.07378	6.287	0.090
14.855	Apigenin	C_15_H_10_O_5_	− 0.39	270.05272	0.689	0.000
14.911	Retusin (flavonol)	C_19_H_18_O_7_	− 0.04	358.10524	2.606	0.054
15.125	S-Curcumene	C_15_H_22_	0.95	202.17234	0.855	0.228
15.185	Fumarin	C_17_H_14_O_5_	− 1.04	298.08381	0.673	0.080
15.266	Salvigenin	C_18_H_16_O_6_	− 0.03	328.09468	0.774	0.000
15.468	Hispidulin	C_16_H_12_O_6_	− 1.08	300.06306	0.298	0.001
15.568	Corchorifatty acid F	C_18_H_32_O_5_	− 0.79	328.22471	11.36	1.870
15.711	Eupatorin	C_18_H_16_O_7_	− 1.05	344.08924	16.75	0.835
15.923	Morphiceptin	C_28_H_35_N_5_O_5_	− 2.06	521.26274	1.881	0.017
16.345	(E)-10-Hydroxydec-2-enoic acid	C_10_H_18_O_3_	− 4.02	186.12485	0.161	0.037
16.410	4-Allyl-2-methoxyphenyl salicylate	C_17_H_16_O_4_	− 0.28	284.10478	0.513	0.003
16.477	10,16-Dihydroxyhexadecanoic acid	C_16_H_32_O_4_	− 1.18	288.22972	2.433	0.020
16.781	Curcumin	C_21_H_20_O_6_	− 0.82	368.12568	0.048	0.193
16.981	3,5-Dihydroxy-4',7-dimethoxyflavone	C_17_H_14_O_6_	− 0.07	314.07902	4.064	0.249
17.008	Glycitein	C_16_H_12_O_5_	− 0.68	284.06828	1.103	0.018
17.705	Kukoamine A	C_28_H_42_N_4_O_6_	− 3.71	530.30847	4.153	0.072
17.839	Ramiprilat	C_21_H_28_N_2_O_5_	− 0.80	388.19951	1.550	0.011
18.023	Asiatic acid	C_30_H_48_O_5_	− 0.86	488.34975	10.55	0.473
18.499	6-O-Methylmangostanin	C_26_H_30_O_7_	− 0.30	454.19902	0.223	0.001
19.152	Curcumene	C_15_H_22_	0.68	202.17229	1.597	0.022
19.641	all-cis-4,7,10,13,16-Docosapentaenoic acid	C_22_H_34_O_2_	− 0.48	330.25572	0.002	0.158
20.895	Stearidonic acid	C_18_H_28_O_2_	− 0.20	276.20888	0.395	0.026
20.898	Oleic acid	C_18_H_34_O_2_	− 0.16	282.25584	0.985	73.14
20.913	Stearic acid	C_18_H_36_O_2_	− 2.48	284.27083	4.281	71.93
21.645	Eicosapentanoic acid	C_20_H_30_O_2_	− 0.29	302.22449	1.495	0.956
21.683	14(Z)-Eicosenoic acid	C_20_H_38_O_2_	− 0.50	310.28703	0.578	31.37
21.800	α-Eleostearic acid	C_18_H_30_O_2_	− 1.05	278.22429	84.11	1.464
22.045	16-Hydroxyhexadecanoic acid	C_16_H_32_O_3_	0.25	272.23521	0.804	0.019
22.051	5b-Cholestane-3a,7a,12a,26-tetrol	C_27_H_48_O_4_	− 0.89	436.35487	0.002	2.216
22.156	Oleanolic acid	C_30_H_48_O_3_	− 0.76	456.36000	2.866	0.361
22.405	Margaric acid	C_17_H_34_O_2_	− 0.59	270.25572	0.003	0.789
22.608	Docosahexaenoic acid	C_22_H_32_O_2_	− 0.50	328.24006	0.264	0.430
22.810	Arachidonic acid	C_20_H_32_O_2_	− 0.54	304.24006	0.429	0.236
23.066	Linoleic acid	C_18_H_32_O_2_	− 1.22	280.23989	66.24	1.174

*WE* water extract, *EE* ethanolic extract, − 5 < accuracy score < 5.

Various compounds present in both water and ethanolic extracts of *O. stamineus* leaves have the ability to prevent oxidation processes, including caffeic acid, rosmarinic acid, etc. (Table 4). Caffeic acid is a member of the phenolic family with good antioxidant properties and works synergistically with other compounds to improve its action; however, it can occasionally act as a prooxidant when consumed in excessive amounts^36^.

Polyphenol caffeic acid (CA), derived from hydroxycinnamic acid, has been claimed as a remedy for many kinds of illnesses, including diabetes^37^. It can reduce blood glucose levels through the inhibition of α-glucosidase and α-amylase. Oboh et al.^38^ discovered that caffeic acid had a superior ability to inhibit α-amylase and α-glucosidase with IC_50_ values of 3.68 µg/mL and 4.98 µg/mL, respectively, and that its activity was better than chlorogenic acid (IC_50_ values for α-amylase and α-glucosidase were 9.10 µg/mL and 9.24 µg/mL, respectively). This is a brief justification of the scientific data supporting the potency of both extracts as antioxidants and α-glucosidase inhibitors.

Although, both extracts contain many compounds or metabolites which can potentially protect against inflammation, as previously reported. For example, kukoamine A is a reported anti-inflammation compound, and is present in both water and 100% ethanolic extracts of *O. stamineus* leaves. This compound inhibits reactive oxygen species (ROS), nitric oxide (NO), prostaglandin E2, cyclooxygenase-2 activity, tumor necrosis factor-α, and interleukin-1 (IL-1), and IL-6 production, according to Wang et al.^39^. The extracts also contain polyunsaturated fatty acids, which are responsible for anti-inflammatory activities. However, the 100% ethanolic extract of *O. stamineus* leaves was more harmful than the water extract of *O. stamineus* leaves. The ethanolic extract is suggested to contain the irritant methyl 4-hydroxycinnamate, which may be responsible for the death of zebrafish larvae.

## Conclusions

The feasibility of use of *O. stamineus* leaves as a herbal treatment requires further investigation. Water extract of *O. stamineus* has interesting potential for use as an α-glucosidase inhibitor and antioxidant agent and is safer for human use compared to the ethanolic extract, with LC_50_ > 800 µg/mL and 21.623 µg/mL, respectively. The activity of water extract of *O. stamineus* leaves can be attributed to several active compounds present in the extract, including fatty acid groups.

## Data Availability

All data for Figs. 1, 2 and 3 and Tables 1, 2, 3 and 4 are provided in the paper.
